# Maternal and Neonatal Outcomes of SARS-CoV-2 Infection in a Cohort of Pregnant Women with Comorbid Disorders

**DOI:** 10.3390/v13071277

**Published:** 2021-06-30

**Authors:** Maria de Lourdes Benamor Teixeira, Orlando da Costa Ferreira Júnior, Esaú João, Trevon Fuller, Juliana Silva Esteves, Wallace Mendes-Silva, Carolina Carvalho Mocarzel, Richard Araújo Maia, Lídia Theodoro Boullosa, Cássia Cristina Alves Gonçalves, Patrícia Pontes Frankel, Maria Isabel Fragoso da Silveira Gouvêa

**Affiliations:** 1 Infectious Diseases Department, Hospital Federal dos Servidores do Estado, Rua Sacadura Cabral, 178, Anexo IV 4° Andar, Rio de Janeiro 20221-161, RJ, Brazil; mlbenamor@hotmail.com (M.d.L.B.T.); trevon@diphse.com.br (T.F.); bebelsgouvea@uol.com.br (M.I.F.d.S.G.); 2 Instituto Nacional de Infectologia Evandro Chagas, Fundação Oswaldo Cruz, Av. Brasil, 4365—Manguinhos, Rio de Janeiro 21040-360, RJ, Brazil; 3 Laboratório de Biologia Molecular, Departamento de Genética, Instituto de Biologia, Universidade Federal do Rio de Janeiro, Av. Carlos Chagas Filho, 373—Sala A1-050—Cidade Universitária da Universidade Federal do Rio de Janeiro, Rio de Janeiro 21941-902, RJ, Brazil; orlandocfj@gmail.com (O.d.C.F.J.); richardmaia.a@hotmail.com (R.A.M.); ldthboullosa@hotmail.com (L.T.B.); cassia.alves@gmail.com (C.C.A.G.); 4 Maternal Fetal Department and Infectious Diseases Department, Hospital Federal dos Servidores do Estado, Rua Sacadura Cabral, 178, Rio de Janeiro 20221-161, RJ, Brazil; drajulianaesteves@gmail.com (J.S.E.); drwallacemendes@yahoo.com.br (W.M.-S.); carolinac.mocarzel@gmail.com (C.C.M.); patricia_frankel@hotmail.com (P.P.F.); 5 Perinatal Health Program, Maternidade Escola, Universidade Federal do Rio de Janeiro, Av. Carlos Chagas Filho, 373—Sala A1-050—Cidade Universitária da Universidade Federal do Rio de Janeiro, Rio de Janeiro 21941-902, RJ, Brazil

**Keywords:** SARS-CoV-2, HIV, obesity, obstetrics, pregnancy

## Abstract

There are some reports and case series addressing Coronavirus Disease 2019 (COVID-19) infections during pregnancy in upper income countries, but there are few data on pregnant women with comorbid conditions in low and middle income Countries. This study evaluated the proportion and the maternal and neonatal outcomes associated with SARS-CoV-2 infection among pregnant women with comorbidities. Participants were recruited consecutively in order of admission to a maternity for pregnant women with comorbidities. Sociodemographic, clinical, and laboratory data were prospectively collected during hospitalization. Pregnant women were screened at entry: nasopharyngeal swabs were tested by RT-PCR; serum samples were tested for IgG antibodies against spike protein by ELISA. From April to June 2020, 115 eligible women were included in the study. The proportion of SARS-CoV-2 infection was 28.7%. The rate of obesity was 60.9%, vascular hypertension 40.0%, and HIV 21.7%. The most common clinical presentations were ageusia (21.2%), anosmia (18.2%), and fever (18.2%). Prematurity was higher among mothers who had a SARS-CoV-2 infection based on RT-PCR. There were two cases of fetal demise. We found a high proportion of COVID-19 among pregnant women with comorbidities. This underscores the importance of antenatal care during the pandemic to implement universal SARS-CoV-2 screening, precautionary measures, and the rollout of vaccination programs for pregnant women.

## 1. Introduction

In 2019, cases of a new respiratory disease caused by a novel Coronavirus emerged in Wuhan, China. The disease, now named Coronavirus disease 19 (COVID-19) is caused by the SARS-CoV-2 virus and rapidly spread worldwide. Since 26 February 2020, when the first case was reported in Brazil, COVID-19 has caused a huge number of infections [1]. On 11 March 2020, COVID-19 was declared a pandemic by the World Health Organization, and is continuing to spread globally. As of June 2021, 180 million cases with more than 3.89 million deaths have been confirmed worldwide. In much of South America, the pandemic is currently spreading out of control. A total of 18.2 million SARS-CoV-2 cases have been reported in Brazil through June 2021, resulting in 507,109,000 deaths [1]. Among these confirmed COVID-19 cases in Brazil, 14,484 have been in pregnant women, 1461 (10.1%) of whom have died [2].

A study of COVID-19 cases reported electronically to the US National Notifiable Diseases Surveillance System analyzed lab-confirmed cases among women of child-bearing age, of whom 8207 were pregnant [3]. Compared to non-pregnant women, non-white pregnant women with underlying medical conditions were 5.4 time more likely to be hospitalized, 1.5 times more likely to be admitted to an intensive care unit (ICU), and 1.7 times more likely to receive mechanical ventilation [3].

COVID-19 screening tests, vaccination, social distancing, mask-wearing, hand hygiene, and avoidance of crowds are among the policies recommended by the WHO and CDC to control the pandemic. According to a systematic review, COVID-19 during pregnancy is associated with increased rates of adverse maternal and neonatal outcomes, particularly in low and middle income countries (LMIC) countries [4]. Universal screening of pregnant women upon admission is already recommended in the United Kingdom, and suggested in other countries such as the United States [5,6]. Allotey et al. in a systematic review reported that 7–12% of pregnant women hospitalized for any reason tested positive for SARS-CoV-2, 62–82% of whom were asymptomatic [7].

The aim of this study was to evaluate the proportion and the maternal and neonatal outcomes associated with SARS-CoV-2 infection in pregnant women with comorbid conditions admitted to a maternal referral center for high-risk prenatal care in Rio de Janeiro.

## 2. Materials and Methods

This was a pilot study motivated by the need to assess possible pregnancy complications during the COVID-19 epidemic. Participants were offered enrollment consecutively in order of admission to the maternity.

This study was conducted at a referral maternity unit for pregnant women with comorbid disorders at Hospital Federal dos Servidores do Estado (HFSE), Rio de Janeiro, a public federal institution funded by the Brazilian Ministry of Health. From 13 April to 17 June 2020, all pregnant women admitted to the maternity unit were invited to participate in this study. The inclusion criteria were: admission to the maternity unit during gestation, ≥18 years of age, willingness to have nasopharyngeal swabs and blood samples collected for diagnosis of SARS-CoV-2 infection, and provided signed informed consent. The exclusion criteria were women not willing to participate and subsequent admissions in the same pregnancy during the study period. Sampling was not longitudinal as most patients received prenatal care at other health institutions, delivered at our hospital, and post-natal follow-up occurred elsewhere.

Sociodemographic, clinical, and laboratory data were prospectively collected during hospitalization using a structured standardized form designed for the study.

We used the Brazilian Diabetes Society criteria for gestational diabetes according to which it is defined as fasting glycemia of 92–125 mg/dL during the first trimester, or 1-h plasma glucose >180 mg/dL or 2-h plasma glucose of 153–199 following a 75 g glucose load during the second and third trimesters [8,9]. This study defined HIV using the following testing algorithm [10]: 1. A plasma sample was tested for HIV-1 by either a chemiluminescence immunoassay (CLIA) (Abbott ARCHITECT HIV Ag/Ab Combo, Abbott Diagnostics, Abbott Park, IL, USA) or an Abbott real time HIV-1 viral load test (Abbott Real-time HIV-1 Abbott Laboratories, Abbott Park, IL, USA). Next, a second sample from the same participant was tested by CLIA or an Abbott. If both samples were positive for HIV-1, the participant was classified as living with HIV.

Nasopharyngeal swabs were systematically collected according to established protocols [11] within 24 h of admission to the maternity. Swabs were refrigerated upon collection and were transported within 2 h to the Molecular Virology Reference Laboratory at Universidade Federal do Rio de Janeiro. Briefly, SARS-CoV-2 RNA from swab media were extracted in the Maxwell MDX Promega automated machine using the Maxwell16 Viral Total Nucleic Acid Purification Kit (Promega, Madison, WI, USA). RT-PCR was standardized in the laboratory using RT-PCR 7500 Thermal Cycler (Applied Biosystems, Foster City, CA, USA) and the Gotaq one-step Probe RT-qPCR System (Pomega). We have strictly followed the CDC protocol [12] which uses the SARS-CoV-2 nucleocapsid targets—N1 and N2—and human RNase P (RP) as control target. After 45 amplification cycles, RT-PCR positive were defined for samples with cycle threshold (ct) below or equal to 38, negative above 40 ct and inconclusive between these ct values. To define the assay limit of detection (LoD) value, we first defined the number of RNA copies of a SARS-CoV-2 isolate by digital PCR. We then made 12 dilutions (a Log2 series) of this viral isolate specimen and eight replicates of each dilution to calculate the LoD by Probit analysis. We figure that the assay has a LoD of 10 copies of viral RNA per 200 μL of swab media. Serum samples were collected within 24 h of admission to the maternity for serology diagnosis of SARS-CoV-2 infection (IgG serology) and processed at Virology Molecular Reference Laboratory (LVM) at Universidade Federal do Rio de Janeiro. The ELISA assay for detection of IgG was developed and validated at the LVM, using the spike protein as antigen, according to protocol described by Perera et al. [13].

Mann–Whitney tests were used for median comparisons, and interquartile range (IQR) intervals were also calculated. Chi-squared or Fisher’s exact tests were used to compare proportions. In parallel, subgroup analysis of only the RT-PCR data was conducted. Associations between SARS-CoV-2 infection and sociodemographic, clinical, and laboratory variables were evaluated. The level of significance was set at 0.05 for all tests. Statistical analysis was performed using SPSS version 20.0. Imbalanced variables (diabetes and ethnicity) were adjusted between the SARS-CoV-2 positive and negative groups using propensity score matching with SAS 9.4.

The study was approved by the institutional review board of the Hospital Federal dos Servidores do Estado (CAAE# 13139720.5.0000.5252, April 2020).

## 3. Results

**Figure 1:**
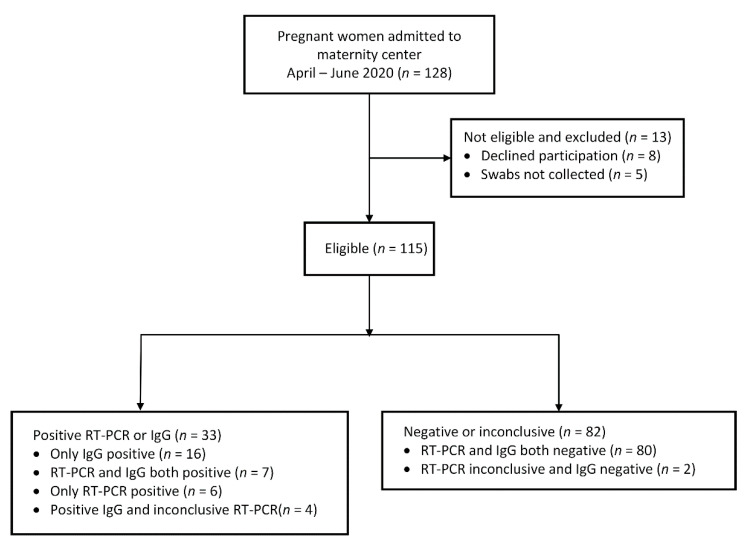
Flowchart of the inclusion and exclusion characteristics of the study population.

From 13 April to 17 June 2020, 128 women were admitted to the HFSE maternity center. Of these 128 women, 13 were excluded: 8 did not agree to participate and 5 women did not have swabs collected. The 115 eligible women were included in the study and had nasopharyngeal swabs for SARS-CoV-2 and plasma collected for serology. See the flowchart for the inclusion and exclusion characteristics of the study population (Figure 1).

Among the 115 women investigated, 28.7% were diagnosed with SARS-CoV-2 infection (33/115). Of the 33 positives, 16 were positive by IgG only, 7 were positive both by IgG and RT-PCR, 6 were positive only by RT-PCR, and 4 were positive by IgG and inconclusive by RT-PCR (Figure 1). In total, 80 tested negative both by RT-PCR and serology, and 2 had inconclusive RT-PCR and negative serology. Those with RT-PCR inconclusive and negative serology or indeterminate serology and negative RT-PCR were classified as negative.

The sociodemographic (Table 1), clinical characteristics and laboratory findings (Table 2), and the maternal and obstetric outcomes (Table 3) are presented below. Both COVID-19 positive and negative pregnant women had similar demographic characteristics (Table 1). The only imbalanced variables were the number of household contacts and diabetes. SARS-CoV-2 positive mothers had an average of 4 household contacts while negative mothers had 3.5 (standardized difference: 0.29). Diabetes was more or less frequent in the SARS-CoV-2 positive women (13.4%) than the SARS-CoV-2 negative ones (25%) (standardized difference: 0.28).

There were 14 participants who were positive by RT-PCR or serology and presented with at least one symptom. The most common clinical presentation was ageusia (7/14), anosmia (6/14), fever (6/14), headache (5/14), dyspnea (5/14), and myalgia (4/14) (Table 2).

The principal maternal comorbid conditions among the participants of the study were obesity 60.9% (70/115), vascular hypertension 40.0% (46/115), HIV 21.7% (25/115), diabetes (gestational diabetes 14.3% (15/105) and type II diabetes 7.0% (8/115)), smoking/tobacco use 5.4% (6/112), alcohol use 4.5% (5/111), use of illicit drugs 1.8% (2/112), rheumatological 0.9% (1/115), and hematological diseases 5.2% (6/115).

We also conducted a separate analysis comparing obstetric outcomes of SARS-CoV-2 RT-PCR positive women to those who were RT-PCR negative, without considering the IgG antibodies. Of 13 women who tested positive for SARS-CoV-2 by RT-PCR, four were asymptomatic when sampled. In a univariate model, gestational age at birth was lower for infants born to mothers who had a SARS-CoV-2 infection based on RT-PCR (*p* = 0.005). In particular, the average gestational age at delivery was 36 weeks for COVID-positive mothers and 38 weeks in negative mothers. All of the 26% of pregnant women with SARS-CoV-2 had preterm deliveries (Table 3). After we performed propensity matching, gestational age at delivery was significantly higher in the group of PCR-positive mothers (*p* = 0.027).

Of the reasons for admission to the maternity center 103 (89.6%) were for delivery, 12 (10.4%) were due to clinical conditions and obstetrical complaints. Among those who delivered during the study, there were 66 vaginal and 36 cesarean births. The principal maternal complications are shown in Table 1, and three pregnant women required admission to the intensive care unit due to oxygen desaturation. There were no maternal deaths.

There was only one case of a malformation, holoprosencephaly, in a mother who was seropositive for SARS-CoV-2. All the pregnant women living with HIV participating in the study were using combined antiretroviral therapy (cART). Among these women, the median CD4+ T cell count was 619 cells/mm^3^. All of them (71% (17/24)) had an undetectable HIV-1 viral load. Concerning neonatal outcomes, there were two cases of fetal demise. One was to an obese woman with SARS-CoV-2 positive serology and negative RT-PCR. The other was to a mother living with HIV, who was RT-PCR inconclusive and had positive serology for SARS-CoV-2. Only the placenta of the woman who was living with HIV was tested and was determined to be RT-PCR-positive for SARS-CoV-2.

## 4. Discussion

The proportion of SARS-CoV-2 in this study population of pregnant women with comorbid conditions at a reference center for high risk gestation was 28.7%. This is higher than the proportion reported in previous studies of pregnant women, which have ranged from 0.56% to 15.4%, and a recent meta-analysis reported a similar range of 7% to 13% [7,14,15,16,17,18,19,20]. This proportion may depend on a variety of factors including the local attack rate, the risk profile of the population investigated and the type of screening assay [21,22,23,24].

The clinical manifestations of COVID-19 in pregnant women in this cohort were largely similar to those of non-pregnant adults in settings with high incidence of SARS-CoV-2 infection, as has been noted in a number of other studies and international guidelines [5,21]. In symptomatic pregnant women, the frequency of fever was similar to that of a multicentric study [25], but lower than in a large CDC registry [26]. This may be explained by the fact that the population of this study were being admitted to our hospital for obstetric and clinical reasons. Three pregnant women were admitted due to signs and symptoms related to COVID-19, all of whom were RT-PCR positive.

Among the principal comorbid conditions in this population were obesity, vascular hypertension, and living with HIV. Obesity has been recognized as one the most important risk factors for severe COVID-19 [27,28]. While in this cohort, obesity was the most frequent comorbid condition, it was not a significant risk factor for SARS-CoV-2 infection.

With respect to neonatal outcomes, in this study the rate of preterm birth among pregnant women with SARS-CoV-2 infection based on PCR tests was 26%, which is higher than the overall rate of preterm birth in Brazil of 11.5% [29]. Concerning malformations, as holoprosencephaly is far more common among diabetic mothers and arises early in gestation, we do not consider it likely that the malformation was associated with maternal SARS-CoV-2 infection. As the sample size of our study was limited, the extent to which these findings can be generalized is unknown. Furthermore, as the site is a reference center for high-risk pregnancies, it is not surprising that preterm births and malformations occurred during the study. It should be noted that a robust review study found no evidence of teratogenicity of SARS-CoV-2 [30].

Coagulation disorders are a concern during pregnancy and can be exacerbated by COVID-19 infection and obesity. However, none of the patients in the cohort experienced complications related to coagulation, perhaps because in this unit, they received antithrombotic prophylaxis as the standard of care for this subpopulation.

There have been few studies of the effects of co-infection of HIV and SARS-CoV-2 in pregnant women. One such study was conducted in South Africa and found that of six pregnant women who were positive for COVID-19 and died, three were living with HIV [31]. In our study, fully 25 (22%) of the study participants were women living with HIV. As the study site is a reference center for HIV in pregnancy, one of the principal comorbidities was HIV. This institution is also a center for prevention of perinatal HIV transmission where testing for HIV is universal during prenatal care and at delivery and cART is the standard of care. Another study in South Africa reported that living with HIV worsened the severity of COVID-19; however, like our study, a number of others have found no evidence of worse clinical outcomes [32,33,34,35]. This population has a higher prevalence of HIV than other populations of pregnant women in which COVID-19 has been investigated, have taken cART as the standard of care with good adherence, had suppressed viral load and high CD4 cell counts. This could partially explain why the severity of COVID-19 among the participants was minimal in our setting.

Universal screening of pregnant women for COVID-19 has been extensively implemented in high income countries experiencing the pandemic. It is widely accepted that in these settings such screening is worthwhile as it can detect asymptomatic cases of COVID-19 [14,36,37]. While in LMICs affected by the COVID-19 pandemic with limited resources, putting universal screening of pregnant women in place will be more difficult. In our view, effort should be made to implement such programs as they can have a positive impact on public health.

Among the strengths of this study is that it was conducted in a maternity for patients with comorbidities and substantial prevalence of HIV, and underscores the importance of establishing governmental policies for pregnant women in LMICs, as has already been implemented in high income countries. These include universal screening, precautionary measures and the rollout of a vaccine program for pregnant women.

Among the limitations of the study was that the follow-up after discharge was limited. In addition, serologic tests for SARS-CoV-2 have a number of limitations, such as false-negative results due to improper timing for collecting a sample for testing or cross-reaction with other Coronaviruses. Additionally, the study did not screen for IgM antibodies.

In conclusion, this study showed high proportion of SARS-CoV-2 infection among pregnant women. This underscores the importance of antenatal care during the pandemic to implement universal SARS-CoV-2 screening, precautionary measures and the rollout of vaccination programs [5,11,38] for pregnant women.

## Figures and Tables

**Table 1:** Baseline demographic and clinical characteristics in a cohort of pregnant women screened for SARS-CoV-2 infection at a prenatal care reference center in Rio de Janeiro, March–June 2020.

Variables		COVID-19		
	All Pregnant Women	Positive	Negative	Standardized Difference
Demographics				
Ethnicity (*n* = 114) (number,%)				
White	33 (28.9%)	11 (34.4%)	22 (26.8%)	0.165
Non-white	81 (71.1%)	21 (65.6%)	60 (73.2%)	
Age at entry (y) (*n* = 114) (median, IQR)	29 (25–35.2)	28 (24–35)	30 (25–36)	0.005
Number of household contacts (*n* = 108) (median, IQR)	4 (3–5)	4 (3–6)	3.5 (3–5)	0.26
Diabetes (gestational or type II) (*n* = 105) (number,%)				
Yes	23 (21.9%)	4 (13.8%)	19 (25.0%)	−0.286
No	82 (78.1%)	24 (86.2%)	57 (75.0%)	
Vascular Hypertension (*n* = 115) (number,%)				
Yes	46 (40%)	13 (39.4%)	33 (40.2%)	−0.016
No	69 (60%)	20 (60.6%)	50 (59.8%)	
Obesity (*n* = 115) (number,%)				
Yes	70 (60.9%)	21 (63.6%)	49 (59.8%)	0.078
No	45 (39.1%)	12 (36.4%)	33 (40.2%)	
Living with HIV (*n* = 115) (number,%)				
Yes	25 (21.7%)	8 (24.2%)	17 (20.7%)	0.084
No	90 (78.3%)	25 (75.8%)	65 (79.3%)	
Tobacco Use during gestation (*n* = 112) (number,%)				
Yes	6 (5.4%)	1 (3.1%)	5 (6.3%)	−0.15
No	106 (94.6%)	31 (96.9%)	75 (93.8%)	
Illicit Drug Use during gestation (*n* = 112) (number,%)				
Yes	2 (1.8%)	1 (3.1%)	1 (1.2%)	
No	110 (98.2%)	31 (96.9%)	79 (98.8%)	0.13
Alcohol use during gestation (*n* = 111) (number,%)				
Yes	5 (4.5%)	1 (3.1%)	4 (5.1%)	−0.1
No	106 (95.5%)	31 (96.9%)	75 (94.9%)	

**Table 2:** Maternal signs and symptoms.

Variables		COVID-19			
	All Pregnant Women	Positive	Negative	Statistical Test	*p*-Value
Signs and Symptoms	All pregnant women (*n* = 115)	Positive (*n* = 33)	Negative (*n* = 82)	*p*-Value Fisher’s Exact Test	
Fever	9 (7.8%)	6 (18.2%)	3 (3.7%)	0.016	
Chills	2 (1. 7%)	1 (3.0%)	1 (1.2%)	0.493	
Headache	16 (13.9%)	5 (15.2%)	11 (13.4%)	0.774	
Dry cough	8 (7.0%)	4 (12.1%)	4 (4.9%)	0.163	
Sore throat	5 (4.3%)	3 (9.1%)	2 (2.4%)	0.141	
Runny nose	6 (5. 2%)	2 (6.1%)	4 (4.9%)	1	
Anosmia	7 (6.1%)	6 (18.2%)	1 (1.2%)	<0.01	
Ageusia	7 (6.1%)	7 (21.2%)	0 (0%)	<0.01	
Persistent pain in the chest	2 (1.7%)	2 (6.1%)	0 (0%)	0.081	
Dyspnea	7 (6.1%)	5 (15.2%)	2 (2.4%)	0.020	
Myalgia	5 (4.3%)	4 (12.1%)	1 (1.2%)	0.023	
Fatigue	3 (2.6%)	3 (9.1%)	0 (0%)	0.022	
Vomiting/ nausea	6 (5.2%)	3 (9.1%)	3 (3.7%)	0.352	
Diarrhea	4 (3.5%)	2 (6.1%)	2 (2.4%)	0.577	

**Table 3:** Obstetrical and neonatal outcomes.

Variables		COVID-19			
	All Pregnant Women	Positive	Negative	Statistical Test	*p*-Value
Obstetrical characteristics and outcomes					
Preeclampsia (*n* = 105) (number,%)				Fisher’s Exact Test	0.227
Yes	15 (14.3%)	2 (6.9%)	13 (17.1%)		
No	90 (85.7%)	27 (93.1%)	63 (82.9%)		
Gestational age at delivery (weeks) (*n* = 99) (median, IQR)	38 (37–39)	38 (35.7–39)	38 (37–39)	Mann-Whitney	0.249
Mode of Delivery (*n* = 102) (number,%)				Pearson Chi-Square	0.956
Vaginal	66 (64.7%)	18 (64.3%)	48 (64.9%)		
C-section	36 (35.3%)	10 (35.7%)	26 (35.1%)		
Neonatal Outcomes (*n* = 102)					
Birth weight (g) (*n* = 100) (number,%)				Fisher’s Exact Test	0.505
<2500	12 (12.0%)	2 (7.4%)	10 (13.7%)		
≥2500	88 (88.0%)	25 (92.6%)	63 (86.3%)		
Preterm delivery (*n* = 99) (number,%)	17 (17.2%)	7 (26.9%)	10 (13.7%)	Fisher’s Exact Test	0.139

## Data Availability

The data presented in this study are available on request from the corresponding author. The data are not publicly available due to privacy restrictions.
